# Surveillance and Risk Analysis for Bovine Babesiosis in England and Wales to Inform Disease Distribution

**DOI:** 10.3390/ani13132118

**Published:** 2023-06-26

**Authors:** Harriet McFadzean, Nicholas Johnson, L. Paul Phipps, Vanessa Swinson, Lisa A. Boden

**Affiliations:** 1 Animal and Plant Health Agency Starcross, Devon EX6 8PE, UK; 2 Vector Borne Diseases, Animal and Plant Health Agency Weybridge, Surrey KT17 3NB, UK; nick.johnson@apha.gov.uk (N.J.); paulphipps10@gmail.com (L.P.P.); 3 Animal and Plant Health Agency Thirsk, North Yorkshire YO7 1PZ, UK; vanessa.swinson@apha.gov.uk; 4 Global Academy of Agriculture and Food Systems, The University of Edinburgh, Midlothian EH25 9RG, UK; lisa.boden@ed.ac.uk

**Keywords:** tick-borne disease, surveillance, *Babesia divergens*, One Health, cattle, epidemiology

## Abstract

**Simple Summary:**

The distribution of ticks is expanding across Europe and the incidence of human and animal tick-borne disease is increasing. This trend is set to continue due to predicted changes in the climate and land use, which alter the interlinked environmental factors required for the feeding and breeding activities of the tick. *Babesia divergens* is a tick-borne piroplasm which poses a major disease threat to cattle in Great Britain and can also cause significant illness in humans. Surveillance of *B. divergens* infection in cattle is currently limited, rendering disease monitoring and mitigation strategies against evolving environmental conditions impossible. This surveillance project collected spatial and temporal data on *B. divergens* infection in British cattle and analysed the risk factors for disease. *B. divergens* was detected in 47.4% of submitted samples. The infections occurred throughout the grazing season. The majority of the cases were diagnosed in South West England; however, other geographical risk areas were identified. These data support future assessment of disease dynamics in the face of evolving environmental conditions. No significant herd- or animal-level disease risk factors were identified. The PCR methods used in this case offer improvements in identifying *Babesia* at species level and offer compatibility with current human diagnostic guidelines.

**Abstract:**

*Babesia divergens* is a zoonotic piroplasm that infects both cattle and humans in Europe. Disease transmission occurs through *Ixodes ricinus* tick bites, a species that is increasing in abundance and distribution across Europe in response to climate and land-use changes. Developments in agri-environment policy and changing consumer demands may also have unintended consequences on tick-borne disease rates. Currently, *B. divergens* surveillance in British cattle is limited, rendering temporal trend analysis and the detection of potential zoonotic hotspots impossible. The objective of this study was to assess syndromic surveillance as a means of determining babesiosis distribution in British cattle, and to evaluate the intrinsic disease risk factors in order to respond to disease threats posed by changing environments. Samples from 95 clinically affected cattle on 70 unique holdings were screened for *Babesia* spp., using established blood smear examination techniques and a *B. divergens*-specific PCR method, between April and December 2021. *B. divergens* was detected in 45/95 animals (47.4%), with PCR offering the advantage of identification at species level. Infection with *Anaplasma phagocytophilum* was detected in 19/95 animals (20%). Co-infection was detected in five animals. The cases were recorded across multiple geographic regions and throughout the sampling period. Univariate logistic regression analysis failed to identify any statistically significant risk factors for *B. divergens* presence. This study demonstrates that bovine babesiosis is geographically widespread throughout England and Wales, placing a large proportion of the cattle population at risk of infection, with the potential for zoonotic transmission to humans.

## 1. Introduction

In Great Britain (GB), bovine babesiosis poses a major disease threat to cattle. This disease, caused by the intraerythrocytic piroplasm *Babesia divergens*, is spread via the bite of the *Ixodes ricinus* tick. The cases traditionally follow a bimodal pattern, with spring and autumn peaks in disease correlating with periods of increased tick activity [1,2]. Clinical signs of disease include pyrexia, malaise, anorexia, and diarrhoea; a more severe presentation includes signs such as constipation, tachycardia, abortion, and aggression [3,4]. At the peak of infection, erythrolysis reaches high levels and animals display characteristic haemoglobinuria, resulting in the condition’s colloquial name, “redwater fever”. Severe production losses and death can occur without treatment [4], particularly in the presence of co-infections with *Anaplasma phagocytophilum*. This zoonotic bacterial organism causes tick-borne fever in cattle, a disease characterised by generalised immunosuppression [5], which is typically mild but can result in pyrexia, reduced milk production and abortion [6]. Secondary infections can arise resulting from the immunocompromised state of infected animals. Tick-borne co-infections such as those with *B. divergens* and *A. phagocytophilum* increase disease morbidity [7,8], and economic consequences in affected herds [9].

In addition to causing disease in cattle, *B. divergens* is classed as an emerging human health threat and has previously resulted in death in a splenectomised British resident [10], and more recently in life-threatening illness in one with no known comorbidities [11]. The prevention of human disease focuses on education and risk mitigation strategies in high-risk areas.

The life cycle and transmission dynamics of *I. ricinus* ticks are linked to environmental factors that include temperature, humidity and the length of the vegetation growing period, which influence tick behaviour [12,13,14]. In response to climate and anthropogenic change [15,16,17,18], tick populations are being affected and, in turn, altering disease transmission dynamics [19]. In response to these evolving conditions, *I. ricinus* is expanding in range across Europe [20,21], and the incidence of tick-borne disease (TBD) is increasing [22,23], leading to increased threats to human and animal health worldwide [24].

Addressing climate change is a major concern of governments across Europe. In GB, agricultural policy is being developed with a reappraised focus on financial incentives to landowners for environmental stewardship measures, delivery of public goods through innovative methods and improving productivity [25]. Changes may also be prompted in response to market pressures, through supplier contract requirements addressing increasing consumer demands for sustainable production methods. The impacts of land-use transformation influencing tick disease dynamics require continued consideration and monitoring.

Predicting and managing the threat of babesiosis is challenging. Tick populations spend the majority of their life cycle in the environment, and a knowledge of temporal disease dynamics at both a local and national level is crucial in mitigating against disease in cattle and humans. Additionally, identifying risk factors for infection at both an intrinsic herd/animal level, and extrinsic environmental level may aid disease mitigation planning on farms. No quantitative research has been conducted in recent decades examining bovine babesiosis prevalence in cattle in England and Wales. Recent literature examining distribution of disease has relied on questionnaire-based studies [26], which may be affected by misdiagnosis of mild or subclinical cases, or animals treated for babesiosis on the basis of clinical signs alone.

Scanning surveillance of livestock diseases in GB is currently passively measured using the APHA Veterinary Investigation Diagnosis Analysis (VIDA) database. This system, developed in 1975, comprises all diagnoses made by the Animal and Plant Health Agency (APHA) and their surveillance partners across GB. However, as similarly reported in Ireland [3], blood sample submissions to government laboratories for bovine babesiosis diagnosis are very low, rendering trend analysis in the face of changing environmental conditions impossible. The widely used diagnostic method of blood smear examination has limitations in both identifying low parasite burdens [27] and classifying *Babesia* at species level [4]. Additionally, there is currently no national-level data sharing of TBD surveillance in GB across public health and veterinary sectors.

The objective of this study was to employ syndromic surveillance to increase temporal and spatial data on bovine babesiosis in cattle in England and Wales, and to analyse herd- and animal-level risk factors of disease. The study findings aim to inform future recommendations on babesiosis surveillance and control in GB whilst examining the potential for a One Health approach to future TBD surveillance and management.

## 2. Materials and Methods

### 2.1. Ethical Statement

The Veterinary Ethical Review Committee of The Royal (Dick) School of Veterinary Studies, University of Edinburgh, approved blood sampling, consent and data handling for this project (Ref. 59/21).

### 2.2. Study Area and Participant Recruitment

An enhanced surveillance project was launched by APHA between April and December 2021 offering free diagnostic blood testing for bovine babesiosis. Prior to and throughout the project, promotional material was circulated to all veterinary practices on APHA mailing lists and through social media to advertise the project. Samples from up to three cattle per farm, displaying clinical signs compatible with babesiosis, could be submitted by private veterinary surgeons. All grazing cattle in counties located in England and Wales were eligible, with no minimum herd size required.

Samples from 95 individual animals from 70 unique farm holdings in England and Wales were received during the sampling period. Most samples were blood-collected from live animals (*n* = 92) and submitted to APHA in tubes coated with ethylenediaminetetraacetic acid (EDTA). However, in three cases, the samples were collected from carcasses submitted for diagnostic post-mortem examination to APHA Veterinary Investigation Centres or partner post-mortem providers. These samples consisted of post-mortem EDTA blood (*n* = 2) and heparinised blood (*n* = 1). All samples were accompanied by a standardised cattle submission form for capturing information on clinical history.

### 2.3. Blood Smear Examination

Thin blood smears were prepared on glass slides and fixed in methanol before staining with 10% Giemsa solution. Stained blood films were examined microscopically under 100× magnification oil immersion objective (Olympus BX40 model BX40F4). Microscopic examination was carried out by trained laboratory personnel following a standard operating procedure. *B. divergens* is a small parasite, which appears as individual intraerythrocytic purple, or red and blue, stained signet-ring-shaped trophozoites, or paired piriform merozoites with a widely divergent angle between them. *A. phagocytophilum* organisms can also be visualised on Giemsa-stained smears and appear as blue intracytoplasmic inclusion bodies in the cytoplasm of granulocytes and monocytes.

### 2.4. Molecular Detection of Blood-Borne Pathogens

Total genomic DNA was extracted from 200 µL of each EDTA blood sample using the DNeasy Blood and Tissue kit (Qiagen, Manchester, UK) as per the manufacturer’s instructions. Total DNA was eluted in 200 µL buffer AE (as provided). Extracted DNA was stored at 4 °C until testing was carried out.

Pan-piroplasm PCR primers were used to screen samples for *Babesia* spp. [28], with inclusion of a TaqMan^®^ probe specific for the *B. divergens* 18S rRNA gene. A real-time PCR was used for the detection of *A. phagocytophilum* [29], a potential co-pathogen in cases of bovine anaemia. Additional information on the primer probes utilized can be found in Table S1.

Amplification was performed in the same tube using the following conditions: heating to 95 °C for 60 s (one cycle), followed by 45 cycles of denaturation via heating to 95 °C for 15 s with subsequent annealing via cooling to 60 °C for 60 s (MX Pro 3000 real-time machine/data analysis using MX3000p v. 4—Agilent Technologies, La Jolla, CA, USA). DNA extracted from a blood sample from a cow previously confirmed as being infected with both *B. divergens* and *A. phagocytophilum* was used as a control throughout the study [30].

A diagnosis of babesiosis was made when either the blood smear examination or the PCR result indicated the presence of *B. divergens* in the blood sample.

### 2.5. Statistical Analysis

Statistical analysis was carried out in R version 4.1.2 and RStudio Version 1.4. Statistical significance was defined as *p* value ≤0.05.

Risk factor variables were selected based on biologically plausible assumptions and data availability, with both farm- and animal-associated risk factors being considered. The following variables were selected: farm region, farm production type, number of cattle on the farm, animal age, sex, breed, number of days the animal had been on the farm, number of previous movements between farms, and whether submitting vets had noted ticks on the animal. Information on these variables, as well as month of submission, clinical signs reported, and mortality was collected on submission forms. The individual animal ear tag number and farm holding number were used to verify data on age, breed, sex, movement records, mortality, and herd size. This was performed using the British Cattle Movement Service Cattle Tracing System database, which is accessible online to registered users [31]. Where data were not provided, or could not be verified, and in the case of missing variable data, submissions were omitted entirely from analysis on a variable-by-variable basis.

Univariate mixed effects logistic regression analysis was performed to assess the relationship between each risk factor variable and a binary outcome using the glmer function with binomial family in the lme4 package in R. The binary outcome was chosen as “*Babesia* spp. detected (yes/no)”, which included diagnoses of both babesiosis as a sole and co-infection. The farm holding number was included as a random effect to account for multiple animals being sampled on single farm holdings. The odds ratio (OR) indicates the odds of infection for each risk factor category when compared to the reference category. The p-values were calculated using the Wald test. In cases of very low sample size where logistic regression analysis was not appropriate, Fisher’s exact test was utilised instead.

Multivariable analysis was considered; however, due to small sample size, this was not deemed possible to perform. For this reason, accounting for potential confounding variables (such as sex and farm type) was not carried out, and it is understood that some reported ORs could differ if investigations had not been constrained to univariate analysis.

## 3. Results

### 3.1. Detection of B. divergens and A. phagocytophilum in Cattle

*B. divergens* and *A. phagocytophilum* PCR testing was completed on all 95 animals. Parallel blood smear examinations were performed in 89 of these. In four submissions, smear examination was not performed due to the sample submitted being unsuitable (autolysis *n* = 3, inappropriate sample type *n* = 1). In two submissions received at the beginning of the project, smear examination was not performed due to limitations in staff resources.

Infection with a tick-borne pathogen was diagnosed in 59/95 animals. Overall, *B. divergens* was detected in 47.4% (45/95) of the animals via PCR and/or smear examination, and *A. phagocytophilum* was detected in 20% (19/95). Five animals were co-infected. At a farm level, cattle from over half of the farms (46/70, 65.7%) were diagnosed with one or more TBDs. Overall *B. divergens* was detected on 57.1% (40/70) of sampled farms, and *A. phagocytophilum* was detected on 22.9% (16/70). The mortality rate for animals infected with *B. divergens* as a sole pathogen was 35% (14/40). For co-infected animals, the mortality rate was 80% (4/5), although this was not statistically significant when compared to that for sole infection with *B. divergens* (Fisher’s exact test *p*-value 0.141) or *A. phagocytophilum* (Fisher’s exact test *p*-value 0.101). No correlation was found between mortality rate and geographical region (Fisher’s exact test, *p*-value 0.195).

Clinical signs were provided for 83 animals, with a total of 21 different signs being reported, all of which could be compatible with clinical babesiosis. For babesiosis cases with clinical signs (*n* = 36), 80.6% reported urinary signs (29/36). Malaise was also a common finding (21/36, 58.3%). For animals with tick-borne fever alone, symptoms were reported in 13 animals. Malaise (11/13, 84.6%) was most commonly reported, with abortion, lameness, anaemia, milk drop, diarrhoea and recumbency also being described in a number of cases. ‘Found dead’ (2/4, 50%), malaise (2/4, 50%), urinary signs (1/4, 25%), anaemia (1/4, 25%) and jaundice and tachycardia (1/4, 25%) were reported in co-infected cattle.

In 44/45 animals where *B. divergens* was detected (as a single or co-infection), the PCR result was positive. In the remaining animals, *Babesia* sp. was detected on slide examination only and was presumed to be *B. divergens* due to the morphological appearance of merozoites or trophozoites.

### 3.2. Seasonal Distribution

**Figure 1:**
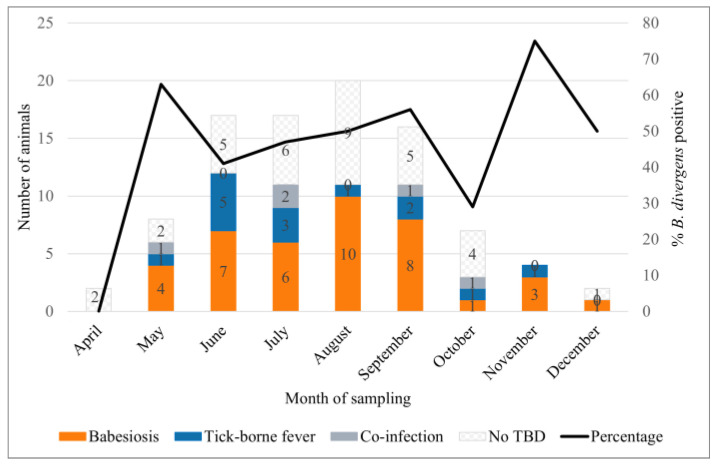
Seasonal distribution and frequency of diagnoses carried out in 95 sampled animals. Line represents proportion of total samples per month that tested positive for *Babesia divergens*.

Diagnoses of babesiosis were carried out in all months between May and December (Figure 1). Diagnoses increased over the summer months before peaking in August and September. An earlier seasonality was observed with tick-borne fever, with over half of diagnoses (9/14, 64.3%) occurring between May and July.

### 3.3. Geographical Distribution

**Figure 2:**
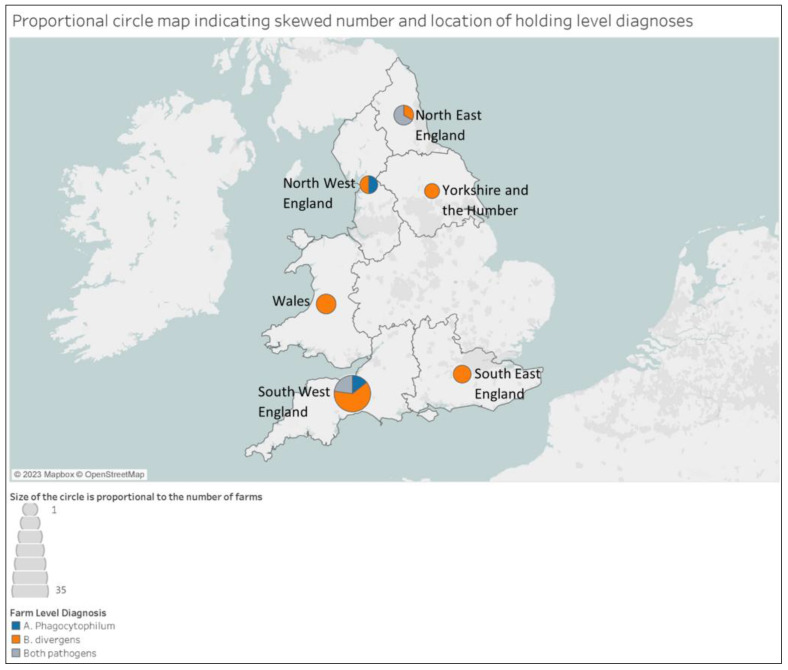
Proportional circle map indicating skewed number and location of holding-level diagnoses. Map generated using software under licence from https://public.tableau.com/app/discover (accessed on 6 January 2023).

*B. divergens* was detected in samples from holdings located in South West England (30/52), Wales (3/4), South East England (2/3), North West England (1/2) Yorkshire and the Humber (1/2) and North East England (3/4). *A. phagocytophilum* was present in samples submitted from holdings located in South West England (13/53), North West England (1/2), and North East England (2/4). All TBD-infected holdings in North East England were located in the county of Northumberland. The majority of the absolute holdings positive for *B. divergens* were located in South West England (Figure 2).

### 3.4. Risk Factor Analysis

No single statistically significant herd- or animal-level risk factors were identified through this study based on the OR and 95% confidence interval (CI) (Table 1 and Table 2).

## 4. Discussion

This study confirmed infection with *B. divergens* on 40 unique holdings within England and Wales. Further cases of *A. phagocytophilum* were detected and a small number of cattle were co-infected.

The seasonality of babesiosis cases in this study confirmed previous concerns regarding changing transmission patterns. In contrast with earlier observations in previous decades in Europe [2,32], we did not find a clear bimodal case distribution. Disease was recorded throughout the grazing season with peak detection, as opposed to an anticipated trough during the summer months. This variation in seasonality signals a need to provide updated disease awareness information to livestock keepers, vets, and health professionals throughout the year. It is possible that the prolonged summer incidence of babesiosis could be attributed to increased awareness of the scheme as advertising progressed. However, recent GB and Irish small-scale studies have also reported potential changes in seasonality [3,9,30]. Long-term variations in climatic conditions across Northern Europe, or a more recent extension to the *I. ricinus* questing season [33,34,35], are both plausible drivers for this changing pattern and warrant a longer-term study to fully examine the seasonality of infection in England and Wales.

The vast majority of project samples were submitted from farms in South West England, and the number of absolute diagnoses of *B. divergens* in the southwest was significantly higher than any other region, corroborating previous questionnaire-based research indicating this to be a high prevalence geographical area [26]. Study findings also identified potential new areas of infection risk. In Northumberland, five diagnoses of TBD were made from three holdings. APHA has not recorded any cases of babesiosis in this county since 2007. This may be due to a variety of reasons including lower farmer engagement involving veterinary/government services, changes in animal management or tick-control practices, or a recent increase in tick disease prevalence in this area. As such, it is not known whether TBDs present an emerging, re-emerging or even endemic disease risk in this area, and further targeted surveillance in this region is needed.

A limitation of this study was the sample size. In spite of free testing being available to all cattle farms in England and Wales, too few samples were received over the sampling period to be able to ascertain an accurate incidence rate, and to conduct a comprehensive risk factor analysis. This study relied on voluntary submission of samples; therefore, some babesiosis cases were likely still diagnosed on history or clinical signs in the absence of diagnostic testing, or samples were examined microscopically by private vets or private veterinary laboratories without resulting in submission to a government laboratory, both of which are known limitations of animal health syndromic surveillance systems. Despite this limitation, the research presented here offers an insight into the current disease dynamics of babesiosis in England and Wales, where no dataset previously existed.

*B. divergens* was the only piroplasm species to be identified in this study, which concurs with the findings of a smaller Scottish study of a similar nature conducted by Gray [36]. In one animal, the PCR result was negative, and babesiosis was diagnosed on the positive blood smear result alone. The TaqMan assay is a highly sensitive and specific method for the identification of *B. divergens*. It cannot be ruled out that the smear examination in this animal was incorrectly positive due to operator error, or that a blood piroplasm was present that was not able to be detected with the molecular methods utilized here. Other piroplasm species that have been shown to infect cattle in Great Britain include *Babesia major* [37,38] and the *Theileria buffeli*/*orientalis* complex [39,40]. However, both species are of low prevalence and low pathogenicity [37,39,41] and would thus likely not result in the clinical signs reported in this study. *Babesia motasi* is also present and has a zoonotic potential but is associated with infection in sheep [38].

Despite there being no evidence of emerging *Babesia* spp. in this study, the emergence of novel *Babesia* sp. has been reported around the world [42,43]. Exotic tick importation to Britain is a continuing threat [44] and changing climatic conditions may in future years sustain populations of non-indigenous tick species, which facilitates pathogen emergence. Although the use of blood smear examination in a practice setting is still warranted to provide a rapid diagnosis in many cases, the ability to accurately identify a wide range of piroplasms through amplicon sequencing at species level is a major advantage of the PCR method. The provision of this PCR test provides private vets with additional testing options and may be especially useful where unexpected mortality/morbidity rates suggest co-infection/emerging *Babesia* spp., or the clinical signs do not corroborate the initial blood smear examination result. Additionally, PCR and smear examination are utilised in GB for human babesiosis diagnosis [11]; thus, by adopting molecular methods within the veterinary sector, surveillance data of humans and cattle could be directly combined and compared, addressing the lack of data standardisation between sectors, which is a major challenge for synthesis of an integrated system for zoonotic diseases [45,46].

Co-infection with both *B. divergens* and *A. phagocytophilum* has previously been demonstrated in GB, with significant detrimental effects on animal health and welfare [9]. In our study, 11.1% of the animals which were *B. divergens*-positive were co-infected with *A. phagocytophilum*, a slightly lower prevalence than the 18% recorded in Sweden by Andersson et al. [7], who suggested that co-infections played a role in disease severity. *A. phagocytophilum* was detected in 20% of animals either as a sole or co-infection. There is little information on the epidemiology of this organism in cattle in England and Wales; however, the presence of clinical signs in all affected animals indicates the need for further studies to quantify the risk to animal health and production this organism poses through further surveillance studies. Beyond tick-borne diseases, other livestock infections could also combine to increase morbidity and mortality within affected animals. Two haemotropic mycoplasmas, *Mycoplasma weyonii* and *Candidatus Mycoplasma haemobos,* have both been previously identified in cattle co-infected with *A. phagocytophilum* in the South West of England using molecular techniques [47]. Similarly, the relationship of these pathogens and their effects on animal morbidity are unknown, although researchers have proposed an increased clinical significance of bovine hemoplasmas [48]. These were not screened for in this study and their presence cannot be ruled out. The number of co-infections diagnosed here was too small for meaningful analysis of mortality rates; however, 4/5 animals died shortly after infection, suggesting again that dual infection increases the severity of disease [7].

Clinical signs reported in cattle infected with *B. divergens* correlated well with those reported in the literature. However, it is of note that haemoglobinuria, reported in 80.6% of babesiosis cases, only presents late in the disease process. The fatality case rate for babesiosis in this study (35%) is markedly higher than the <10% reported following treatment by Jerram and Willshire [49] and was not linked to farm region. The sampling method we utilised potentially attracted submissions of cases with high parasitaemia that were refractory to treatment. However, poor detection of early disease was a contributory factor for increased mortality rate in an Irish study [3]. Considering the consequences of cattle mortality on animal welfare and “unproductive greenhouse gas emissions” [50], there is an urgent need for renewed awareness campaigns on disease detection amongst vets and farmers across Britain.

No significant intrinsic risk factors for babesiosis infection were identified through this study. The relatively small sample size, which is a recognized limitation of this study, means that the lack of statistically significant associations detected between risk factors and disease outcomes may both be a result of there being no association, or there being insufficient power to detect the association. Despite this, some meaningful results have been obtained. Although not statistically significant, females, beef cattle, and cattle that had previously moved holdings were all deemed to be potentially meaningful factors based on both biological assumptions, and the calculated OR and 95% CI, which corroborates previous work describing babesiosis risk factors. It has previously been suggested that beef herds have a higher prevalence of *B. divergens* infection due to the higher likelihood of grazing marginal land with increased suitability for *I. ricinus* survival [51]. Less frequent inspection of beef cattle compared to dairy cattle may also influence the likelihood of animals encountering tick burdens that are left untreated. A drop in milk yield for dairy cows is also an indicator that might signal early TBD. The movement of animals has been previously considered a major risk for *B. divergens* infection [30,32], and the introduction of naïve animals into areas with *Babesia* sp.-infected ticks represent a particularly high risk for disease [9]. Animal movements between different holdings, even if an animal has built previous age-related inverse immunity, could change tick exposure and lead to the waning or complete absence of immune protection [26]. The significance of sex is unclear. Many female physiological processes such as calving and lactation can have immunosuppressive effects, leading to this sex potentially being at greater odds of disease [52]. Indeed, general positive health and nutrition statuses were found to be advantageous in preventing babesiosis by He et al. [43]. Both the low sample size of males and the potential for confounding are important limiting factors in the interpretation of the importance of this variable. The effect of sex on disease status requires further investigation.

Extrinsic factors are likely to be more influential regarding infection risk in both cattle and humans due to the significant effects these have on tick life cycles, and may have a role in differing geographical prevalence of disease. For example, upland habitat [26], the presence of red deer [53], fragmented forest sites (with oak as opposed to pine forests) [54], and field margin ecotones [19] have all been linked to increased tick abundances. In this study, extrinsic disease factors were not investigated due to a lack of integration between environmental and livestock disease data systems. A robust framework of comparing climate, land use, vegetation growth and host availability, alongside disease trends, would provide a more holistic approach to veterinary and human disease surveillance, identification of risk factors and prediction of seasonality in future years. However, vast knowledge gaps exist surrounding tick ecology, and implementing a One Health approach requires significant political, institutional, and financial commitment, which necessitates time and investment. A modelling approach to predicting disease risk on a regional level, and further studies focusing on extrinsic factors relating to potential changes in agricultural policy pose cost-effective short-term alternatives for consideration.

## 5. Conclusions

This study has demonstrated that the addition of syndromic surveillance can increase the detection of babesiosis in England and Wales, and can support future assessment of changes to TBD dynamics in the face of changing climatic conditions and land-use transformation. Risk factor analysis has not indicated any significant intrinsic risk factors that may inform disease mitigation; however, larger studies and deeper investigation of extrinsic factors are required. The value of molecular testing methods has been demonstrated through the definitive identification of the infecting *Babesia* species. The results of this study have already informed [55], and will continue to inform awareness campaigns and contribute to the evidence base considered for government surveillance policy in Great Britain.

## Figures and Tables

**Table 1:** Univariate analysis of farm-level variables for presence of *Babesia* sp. in 95 animals with farm holding number as a cluster variable.

Variable	Total Animals	*Babesia*-Positive (%)	Estimate	Standard Error	*p* Value	Odds Ratio (OR)	95% Confidence Interval (CI)
Region (*n* = 95)							
South West England	76	34 (44.7)	-	-	-	-	-
Rest of England and Wales	19	11 (57.9)	0.54	0.556	0.33	1.71	0.58–5.09
Farm type (*n* = 94)							
Dairy	37	13 (35.1)	-	-	-	-	-
Beef	57	32 (56.1)	1.07	0.576	0.06	2.91	0.94–8.99
Number on holding (*n* = 95)							
0–99	23	13 (56.5)	-	-	-	-	-
100–199	24	14 (58.3)	0.06	0.625	0.92	1.06	0.31–3.61
200–299	17	8 (47.1)	−0.42	0.691	0.54	0.66	0.17–2.54
300–399	15	4 (26.7)	−1.35	0.794	0.09	0.26	0.05–1.23
>400	16	6 (37.5)	−0.83	0.723	0.25	0.44	0.11–1.81

**Table 2:** Univariate analysis of animal-level variables for the presence of *Babesia* sp. in 95 animals with farm holding number as a cluster variable.

Variable	Total Animals	*Babesia*-Positive (%)	Estimate	Standard Error	*p* Value	Odds Ratio (OR)	95% Confidence Interval (CI)
Age (*n* = 82)							
Age (<2 years)	14	6 (42.8)	-	-	-	-	-
Age (2–5 years)	27	15 (55.5)	0.53	0.722	0.46	1.70	0.41–7.01
Age (>5 years)	41	21 (51.2)	0.31	0.680	0.65	1.36	0.36–5.17
Sex (*n* = 93)							
Female	81	42 (51.9)	-	-	-	-	-
Male	12	3 (25)	−1.17	0.708	0.10	0.31	0.08–1.24
Breed (*n* = 89)							
Pedigree	58	29 (50)	-	-	-	-	-
Crossbreed	31	15 (48.4)	−0.07	0.467	0.89	0.94	0.37–2.34
Days on farm (*n* = 82)							
<365 days	25	15 (60)	-	-	-	-	-
>365 days	57	27 (47.4)	−0.54	0.524	0.31	0.58	0.21–1.63
Number of moves (*n* = 83)							
0	46	19 (41.3)	-	-	-	-	-
≥1	37	23 (62.2)	0.92	0.522	0.08	2.51	0.90–6.98
Ticks reported (*n* = 95)							
Yes	18	8 (44.4)	-	-	-	-	-
No	77	37 (48.1)	0.11	0.576	0.84	1.12	0.36–3.46

## Data Availability

The data presented in this study are not publicly available due to privacy issues regarding identifiable information of animal owners. The R code is available on request from the corresponding author.

## Supplementary Materials

The following supporting information can be downloaded at: https://www.mdpi.com/article/10.3390/ani13132118/s1, Table S1: Primers utilised for dual testing of bovine blood samples based on the primer probe combinations by Armstrong et al. [28] and Courtney et al. [29], with inclusion of a TaqMan^®^ probe specific for the *B. divergens* 18S rRNA gene.
