# Supplementary material for: Surveillance and Risk Analysis for Bovine Babesiosis in England and Wales to Inform Disease Distribution
Source: Animals (Basel). 2023 Jun 26;13(13):2118. doi: 10.3390/ani13132118 (PMC10339932; doi:10.3390/ani13132118)
Supplement: Supplementary file 1 [file animals-13-02118-s001.zip › animals-2393848-supplementary.pdf]

Table S1. Primers utilised for dual testing of bovine blood samples based on the primer probe combinations by Armstrong et al. [28] and Courtney et al. [29], with inclusion of a TaqMan® probe specific for the *B. divergens* 18S rRNA gene.

Pathogen	Primer name	Primer Sequence	Reaction mix	Product size
Anaplasma phagocytophilum	ApMSP2f	ATGGAAGGTAGTGTTGGTTATGGTATT	1 µM	77 base pair
	ApMSP2r	TTGGTCTTGAAGCGCTCGTA	1 µM	
	ApMSP3p (Hex)	TGGTGCCAGGGTTGAGCTTGAGATTG	0.2 µM	
Babesia spp.	Piro A	AATACCCAATCCTGACACAGGG	0.8 µM	407 base pair
	Piro B	TTAAATACGAATGCCCCCAAC	0.8 µM	
	BdivProbe (Fam)	5′-FAM-TGCGTGGTGTTAATATTGACTAATGTCG-TAMRA-3′	0.2 µM	
